# Supplementary material for: WordCommentsAnalyzer: A windows software tool for qualitative research
Source: F1000Res. 2018 Sep 4;7:536. Originally published 2018 May 3. [Version 2] doi: 10.12688/f1000research.14819.2 (PMC6137411; doi:10.12688/f1000research.14819.2)
Supplement: Supplementary file 3 [file f1000research-7-17614-s0002.tgz › b2a79496-4ada-47c4-9682-a99ce867836d.zip!/Eight Iranian Health Journals/Archives of Rehabilitation_7.docx]

© Archives of Rehabilitation (RJ). University of Social Welfare and Rehabilitation Sciences, Koodakyar Alley, Daneshjoo Blv., Evin, Tehran, Iran. This work is licensed under [CC BY-NC 4.0](https://creativecommons.org/licenses/by-nc/4.0/).

Volume & Issue: دوره 17 - شماره 3

Title: مقایسه مشارکت کودکان فلج مغزی 4 تا 6 سال با کودکان همسال طبیعی در کاره‌ها

http://rehabilitationj.uswr.ac.ir/article-1-2076-fa.html

هدف کاره به فعالیت‌های مربوط به مراقبت از خود، مشارکت در منزل، بازی، فعالیت‌های اوقات فراغت، مشارکت اجتماعی و محیط‌های آموزشی گفته می‌شود. مشارکت کودکان در کاره‌ها با سلامت و رفاه آن‌ها ارتباط دارد. هدف از مطالعه حاضر، مقایسه مشارکت کودکان فلج مغزی 4 تا 6 سال درمقایسه‌با کودکان همسال در هریک از کاره‌ها طبیعی بوده است. روش بررسی در این مطالعه مورد-شاهد، 50 کودک مبتلا به فلج مغزی با دامنه سنی 4 تا 6 سال از کلینیک‌های خصوصی جسمی‌حرکتی و 50 کودک سالم دامنه سنی 4 تا 6 سال از پیش‌دبستانی‌ها و مهدکودک‌های سطح شهر تهران در سال 1394 در مطالعه شرکت کردند. نمونه‌های نام‌برده به روش غیراحتمالی آسان و دردسترس انتخاب شدند. مشارکت این کودکان از طریق نسخه فارسی پرسش‌نامه مشارکت کودکان (CPQ) ارزیابی شد. پس‌از ارائه توضیحات لازم والد کودک پرسش‌نامه مذکور را تکمیل کرد. برای تجزیه داده‌ها از نسخه 18 نرم‌افزار SPSS و به‌منظور مقایسه مشارکت کودکان فلج مغزی و سالم در کاره‌ها از آزمون تی مستقل استفاده شد. یافته‌ها میانگین سنی کودکان مبتلا به فلج مغزی و سالم در این مطالعه به‌ترتیب 5 و 5 سال و یک ماه بود. از نظر تنوع، شدت، میزان کمک، میزان لذت و رضایت والدین، مشارکت در حیطه‌های مراقبت از خود، مشارکت در منزل، بازی، اوقات فراغت و محیط‌های آموزشی تفاوت معناداری بین کودکان فلج مغزی و کودکان سالم همسال مشاهده شد (001/0P<). در حیطه مشارکت اجتماعی تفاوت معناداری بین دو گروه نبود (05/0P>). نتیجه‌گیری نتایج نشان داد میزان مشارکت کودکان مبتلا به فلج مغزی در کاره‌ها پایین‌تر از کودکان سالم است؛ بنابراین آگاهی از مشارکت این کودکان به متخصصان سیستم بهداشتی برای طراحی درمان‌های متناسب با نیازهای کودکان و ارتقای مشارکت و سلامت آن‌ها کمک می‌کند. احتمالاً یکی از دلایل پایین‌بودن میزان مشارکت کودکان فلج مغزی در کاره‌ها هدفمندنبودن بسیاری از مداخلات و استفاده‌نکردن از کاره‌­ها در مداخلات است.

Title: اثربخشی بازی‌درمانی گروهی بر ارتباط کودکان 5 تا 8 سال مبتلا به اختلال اتیسم با عملکرد بالا

http://rehabilitationj.uswr.ac.ir/article-1-2077-fa.html

هدف یکی از ملاک‌های تشخیصی اختلال اتیسم، آسیب به مهارت‌های ارتباطی فرد است که مشکلاتی را در فراگیری مهارت‌های زبانی و گفتاری به‌وجود می‌آورد. امروزه استفاده از بازی‌درمانی با هدف حمایت از کودکان با اختلال اتیسم رواج یافته است. بازی‌درمانی رویکردی فعال است که به کودک اجازه می‌­دهد تا احساسات هشیار و ناهشیار خود را به‌واسطه بازی آشکار کند. این مطالعه با هدف تعیین اثربخشی بازی‌درمانی گروهی بر ارتباط کودکان 5 تا 8 سال مبتلا به اختلال اتیسم با عملکرد بالا انجام شد. روش بررسی پژوهش حاضر مطالعه‌ای آزمایشی با طرح پیش‌آزمون‌پس‌آزمون با یک گروه کنترل بود. از جامعه آماری کودکان پسر 5 تا 8 ساله مبتلا به اختلال اتیسم، 12 کودک مراجعه‌کننده به بنیاد خیریه کودکان اتیسم در سال 1392، به‌شیوه هدفمند انتخاب شدند. معیارهای ورود کودکان به مطالعه شامل این نکات بود: تشخیص اختلال اتیسم با عملکرد بالا، باسوادبودن والدین برای تکمیل پرسش‌نامه، مبتلانبودن کودک به اختلال‌های بینایی و شنوایی و جسمی‌حرکتی مانند فلج مغزی و حضورنداشتن در جلسات مداخله بازی‌درمانی مشابه به‌طورهم‌زمان. کودکان به‌صورت تصادفی در دو گروه آزمایش و کنترل (هر گروه 6 نفر) قرار گرفتند. گروه آزمایش افزون‌بر خدمات آموزشی بنیاد خیریه (شرکت در جلسات کاردرمانی ذهنی و جسمی و گفتاردرمانی)، در 20 جلسه 45 تا 60 دقیقه‌ای (3 جلسه در هفته) بازی‌درمانی گروهی شرکت کردند؛ ولی گروه کنترل فقط خدمات آموزشی و درمانی روزانه بنیاد خیریه را دریافت کردند. افراد هر دو گروه از نظر مهارت‌های ارتباطی قبل و بعد از بازی‌درمانی گروهی و 2 ماه پس از آن، با استفاده از مقیاس رتبه‌بندی اوتیسم گیلیام (گارس) ارزیابی شدند. داده‌های به‌دست‌آمده از مرحله پیش‌آزمون و پس‌آزمون با استفاده از تحلیل کوواریانس تجزیه‌وتحلیل شد. به‌منظور بررسی پایداری اثر مداخله (2 ماه پس از آخرین جلسه بازی‌درمانی) آزمون اندازه‌گیری مکرر به‌کار رفت. یافته‌ها نتایج تحلیل کوواریانس نشان داد که بازی‌درمانی گروهی بر ارتباط کودکان مبتلا به اختلال اتیسم با عملکرد بالا تأثیر معنادار می‌گذارد. همچنین نتایج آزمون اندازه‌گیری مکرر بیانگر آن بود که این تأثیر پس از 2 ماه پیگیری باقی می‌ماند (001/0>P). نتیجه‌گیری از پژوهش حاضر نتیجه گرفته می‌شود که بازی‌درمانی گروهی به‌عنوان فعالیتی با قابلیت انطباق‌پذیری درخور توجه، نوعی وسیله برای درک و برقراری ارتباط با کودک به‌شمار می‌رود و نتایجی از این دست را در کودک به‌دنبال دارد: 1. در برقراری ارتباط با دیگران از نمادهای غیرکلامی استفاده کند؛ 2. ادراکش را گسترش دهد؛ 3. اضطراب کمتری را تجربه کند؛ 4. با کاوش درزمینه تعارض‌های پیرامون خود احساسات خود را بروز دهد؛ 5. خلأ بزرگ بین تجارب و ادراک خود را برطرف کند و از این طریق بتواند به بینش و حل‌مسئله دست یابد. در بازی‌درمانی گروهی کودک شیوه همکاری و تبادل با دیگران و رعایت حقوق آن‌ها را می‌آموزد و طبیعی به‌نظر می‌رسد که کودک بتواند در مقابل کمک‌خواهی دیگران پاسخگو باشد و به‌هنگام بروز مشکلات در حین بازی، بردباری و صبوری نشان دهد و از این طریق، مهارت ارتباط را در خود ارتقا بخشد. بنابراین بازی به‌عنوان یک روش آموزشی و درمانی مناسب برای ارتقای مهارت‌های ارتباطی کودکان با اختلال اتیسم به‌شمار می‌­رود.

Title: تعیین اولویت نیازهای آموزش هندلینگ مادری به مراقبان خانوادگی کودکان مبتلا به فلج مغزی براساس رویکرد خانواده‌محور

http://rehabilitationj.uswr.ac.ir/article-1-2078-fa.html

هدف مراقبان خانوادگی کودکان مبتلا به فلج مغزی در سیر مراقبت از کودک فلج مغزی با مشکلات مراقبتی در حیطه‌های مراقبت فردی، پی‌جویی برای درمان، تطابق‌های مراقبت آموزشی و اوقات فراغت (بازی‌وتفریح) مواجه می‌شوند. آن‌ها به آموزش‌های ویژه‌ای نیاز دارند تا بتوانند نیازهای خود را در حین مراقبت برطرف کنند. هدف از این مطالعه تعیین اولویت نیازهای آموزش هندلینگ مادری در مراقبان خانوادگی کودکان مبتلا به فلج مغزی براساس رویکرد خانواده‌محور بود. روش بررسی این پژوهش از نوع توصیفی‌تحلیلی بود که به‌صورت مقطعی انجام شد. جامعه هدف مطالعه حاضر کودکان فلج مغزی 4 تا 12 سال شهر اراک و نمونه‌های دردسترس کودکان مراجعه‌کننده به مراکز توانبخشی بودند. از بین این کودکان 186 کودک که شرایط ورود به مطالعه را داشتند، به‌صورت ساده و از نمونه‌های دردسترس انتخاب شدند. برای تعیین سطح عملکرد حرکتی ظریف از سیستم طبقه‌بندی توانایی دستی (MACS) و برای تعیین اولویت نیازها از مقیاس عملکرد کاری کانادایی (COPM) استفاده شد. داده‌های به‌دست‌آمده با استفاده از آماره‌های توصیفی و روش تحلیلی واریانس دوطرفه تجزیه‌و‌تحلیل شد.  یافته‌ها در این پژوهش 89 دختر و 97 پسر مبتلا به فلج مغزی، با میانگین سنی 52/7 و انحراف‌معیار 38/2 شرکت داشتند که براساس نوع فلج مغزی 67/7درصد اسپاستیک، 13/9درصد دیس‌کنیتیک، 5/9درصد آتاکسیک و 4/8درصد هیپوتونیک بودند. بیشترین اولویت نیاز آموزش هندلینگ مادری، آموزش در حیطه مراقبت از خود و مربوط به سطح پنج سیستم طبقه‌بندی توانایی دستی بود. اولویت نیازهای آموزش هندلینگ مادری به مراقبان خانوادگی کودکان مبتلا به فلج مغزی براساس عملکرد کاری (مراقبت از خود و بهره وری و بازی‌وتفریح) در سطوح عملکرد حرکتی ظریف، مربوط به سرویس بهداشتی و مربوط به سطح چهارم بود. بین اولویت نیازهای آموزش هندلینگ مادری کودکان مبتلا به فلج مغزی از نظر سن و سطح عملکرد حرکتی ظریف و سن و بهره‌وری و سن و تفریح تفاوت معنی داری وجود نداشت (05/0P>). درزمینه سن و مراقبت از خود، این تفاوت آماری معنی‌دار شد (05/0P<)؛ یعنی با تغییر وضعیت از نظر سن در سطوح توانایی عملکرد دست و بهره‌وری و تفریح، از نظر نوع نیازهای آموزشی هندلینگ مادری تفاوت آماری معنی‌داری به‌وجود نمی‌آید. باوجوداین درباره مراقبت از خود این تفاوت آماری معنی‌دار است. نتیجه‌گیری نتایج نشان داد بیشترین اولویت نیاز آموزش هندلینگ مادری در حیطه مراقبت از خود، غذادادن، سرویس بهداشتی، تحرک و جابه‌جایی است. از طرفی هرچه سطح عملکرد توانایی دستی بیشتر می‌شود، میزان نیازهای آموزشی هندلینگ مادری برای مراقبان افزایش و با افزایش سن کودکان مبتلا به فلج مغزی این اولویت‌ها تغییر می‌یابند. از طرفی تعامل هم‌زمان سن و سطح عملکرد توانایی دستی کودک مبتلا به فلج مغزی تأثیری بر اولویت نیاز آموزشی هندلینگ مادری ندارد. آموزش نظام‌مند خانواده‌ها و مراقبان درزمینه نیازهای هندلینگ مادری کودک فلج مغزی و نحوه صحیح برطرف‌کردن آن‌ها و چگونگی مراقبت از این کودکان گامی تسهیل‌کننده درراستای ارتقای آموزش هندلینگ مادری کودک مبتلا به فلج مغزی است. به‌نظر می‌رسد شناخت و آموزش هندلینگ مادری برای کاردرمانگرانی که هم نقش درمان کودک فلج مغزی و هم نقش آموزش به خانواده را برعهده دارند، بتواند راهگشای مراقبت و درمانی اثربخش باشد.

Title: بررسی ویژگی‌های آکوستیکی مربوط به کنترل حرکتی گفتار در کودکان لکنتی و غیرلکنتی

http://rehabilitationj.uswr.ac.ir/article-1-2080-fa.html

هدف لکنت رشدی، اختلالی در روانی گفتار است که توسط تکرارهای غیرارادی، کشیده‌گویی و گیر بدون صدا به‌ویژه در بخش اول سخن تشخیص داده می ‌شود و تاکنون علت قطعی بروز آن مشخص نشده است. یکی از نظریات مطرح‌شده در این زمینه، فرضیه نقص در کنترل حرکتی گفتار است که در آن کنترل، زمان‌بندی و هماهنگی عضلات بین مسیرهای تنفسی و حنجره‌ای و راه صوتی گفتار آسیب می ‌بیند. فرکانس پایه، شدت صوت، دامنه تغییرات فرکانس پایه، دامنه تغییرات شدت صوت و زمان شروع آواسازی ازجمله مهم‌ترین مؤلفه‌های آکوستیکی است که معمولاً برای ارزیابی غیرمستقیم عملکردهای فیزیولوژیکی زیربنایی عملکردهای کنترل حرکتی گفتار استفاده می‌شوند. هدف از انجام این مطالعه، بررسی برخی از ویژگی‌های آکوستیکی کنترل حرکتی گفتار در کودکان مبتلا به لکنت و غیرلکنتی دوزبانه آذری و فارسی و غیرلکنتی تک‌زبانه فارسی است. روش بررسی این پژوهش به‌صورت توصیفی‌تحلیلی و از نوع بررسی مقطعی‌مقایسه‌ای انجام شد. نمونه‌های مطالعه‌شده، 25 پسر مبتلا به لکنت دوزبانه و 23 پسر غیرلکنتی دوزبانه و 21 پسر غیرلکنتی تک‌زبانه در محدوده سنی 6 تا 10 سال بودند. مؤلفه‌های آکوستیکی فرکانس پایه، شدت صوت، دامنه تغییرات شدت صوت، دامنه تغییرات فرکانس پایه و زمان شروع آواسازی در تکالیف کشیدن واکه‌های /a/ و /i/ و همچنین تکرار عبارات حامل و با استفاده از نرم‌افزار PRAAT در آزمودنی‌ها ارزیابی و تحلیل شد. اطلاعات جمع‌آوری‌شده و مقادیر به‌دست‌آمده با استفاده از نسخه 17 نرم‌افزار SPSS مشخص شد. تجزیه‌وتحلیل داده‌های به‌دست‌آمده برای مقایسه گروه‌های لکنتی و غیرلکنتی دوزبانه و تک زبانه با استفاده از آزمون‌های تحلیل واریانس یک‌راهه و کروسکال‌والیس براساس اهداف مطالعه انجام شد. یافته‌ها مطالعه حاضر به‌منظور بررسی ویژگی‌های آکوستیکی مؤثر در کنترل حرکتی گفتار در هرسه گروه افراد لکنتی و غیرلکنتی دوزبانه و تک‌زبانه انجام شد. تجزیه‌وتحلیل ویژگی‌های آکوستیکی مطالعه‌شده شامل فرکانس پایه، شدت صوت، دامنه تغییرات شدت صوت، دامنه تغییرات فرکانس پایه و زمان شروع آواسازی در تکالیف کشیدن واکه‌های /a/ و /i/ و همچنین تکرار عبارات حامل، حاکی‌از نبودِ تفاوت معنادار بین گروه لکنتی دوزبانه و غیرلکنتی دوزبانه و تک‌زبانه بود (P>0/05).  نتیجه‌گیری با استناد به یافته‌های این پژوهش می‌توان چنین استنباط کرد که ویژگی‌های کنترل حرکتی گفتار مطالعه‌شده در این تحقیق شامل فرکانس پایه، شدت صوت، دامنه تغییرات فرکانس پایه، دامنه تغییرات شدت صوت و زمان شروع آواسازی در گفتار روان کودکان مبتلا به لکنت و غیرلکنتی و بین گروه های زبانی تفاوت معناداری ندارد. تاکنون مطالعات متعدی نشان داده‌اند که لکنت ارتباط نزدیکی با نقص در کنترل حرکتی گفتار دارد. باتوجه‌به این موضوع که در بیشتر مطالعات تصویربرداری عصبی، تفاوت‌های ساختاری و عملکردی مغزی مربوط به کنترل حرکتی گفتار افراد لکنتی مشاهده شده ‌است، در‌صورتی‌که نتایج مطالعه حاضر نتوانست این تفاوت‌های رفتاری را نشان دهد. یافته‌های این پژوهش مبنی‌بر وجودنداشتن تفاوت معنادار در متغیرهای مطالعه‌شده، از فرضیه وجود محدودیت‌های کنترل مهارت‌های حرکتی گفتار در کودکان لکنتی حمایت نمی‌کند.

Title: طراحی و تعیین روایی و پایایی پرسش‌نامه رضایت والدین از خدمات ارائه‌شده به کودکان کم‌شنوا

http://rehabilitationj.uswr.ac.ir/article-1-2081-fa.html

هدف سنجش رضایت والدین از خدمات ارائه‌شده به کودکان کم‌شنوا، از شاخص‌های مهم ارزیابی اثربخشی برنامه‌های مراکز خانواده و کودک کم‌شنوا و همچنین ابزار مفیدی برای بررسی نقایص این برنامه‌هاست. براین‌اساس در مطالعه حاضر طراحی و تعیین روایی و پایایی پرسش‌نامه رضایت والدین از خدمات ارائه‌شده به کودکان کم‌شنوا زیر 6 سال مدنظر بوده است. روش بررسی پژوهش حاضر مطالعه‌ای توصیفی‌تحلیلی و از نوع اعتبارسنجی و ابزارسازی بود. پرسش‌نامه محقق‌ساخته رضایت‌مندی والدین از خدمات ارائه‌شده به کودکان کم‌شنوا شهر تهران توسط 60 نفر از والدین کودکان کم‌شنوا تکمیل شد. این افراد به‌طورتصادفی انتخاب شده و حداقل یک‌سال از خدمات آموزشی و توانبخشی شنوایی مراکز خانواده و کودک کم‌شنوا شهر تهران استفاده کرده بودند. 15 نفر از این والدین به‌منظور سنجش تکرار‌پذیری در آزمون مجدد شرکت کردند. این پرسش‌نامه از 5 زیرآزمون تشکیل شده بود. این زیرآزمون‌ها عبارت بود از: رضایت از امکانات ساختاری و محیطی مرکز ( شامل 7 سؤال)، رضایت از شرایط بهداشتی مرکز (شامل 10 سؤال)، رضایت از خدمات و عملکرد مرکز (شامل 18 سؤال)، رضایت از ارتباط و شیوه برخورد کارکنان مرکز (شامل 12 سؤال) و رضایت از هزینه‌های پرداختی در مقابل خدمات (شامل3 سؤال). به‌منظور اعتبارسنجی و خصوصیات روان‌سنجی پرسش‌نامه طراحی‌شده این دست گزینه‌ها ارزیابی شد: رضایت والدین از خدمات ارائه‌شده به کودکان کم‌شنوا، ثبات درونی در سطح مورد و مقیاس، همبستگی مورد با کل مقیاس، تکرارپذیری آزمون‌بازآزمون و روایی صوری و روایی محتوایی. درنهایت، داده‌های موجود با استفاده از نسخه 17 نرم‌افزار SPSS تجزیه‌وتحلیل آماری شد. روش‌های آماری مطالعه حاضر عبارت بود از: محاسبه ضریب‌های آلفای کرونباخ، همبستگی اسپیرمن و آزمون‌های کولموگروف‌اسمیرنوف، فریدمن،کروسکال‌والیس و یومان‌ویتنی.  یافته‌ها درباره ویژگی‌های روان‌سنجی روایی محتوایی پرسش‌نامه 79/0 و پایایی ثبات درونی 90درصد به‌دست آمد. همبستگی مورد با کل مقیاس برای هر مورد محاسبه شد که همگی موارد، همبستگی مناسبی را نشان داد. به‌علاوه پایایی آزمون‌بازآزمون 75درصد بود. در این بررسی بیشتر والدین از تمامی حیطه‌های خدماتی مراکز راضی یا نسبتاً راضی بودند. این حیطه‌ها شامل این نکات بود: رضایت‌مندی کلی (5/69درصد)، امکانات ساختاری و محیطی (66/80درصد)، شرایط بهداشتی (75درصد)، خدمات و عملکرد مرکز (91/66درصد)، ارتباط و شیوه برخورد کارکنان (91/66درصد) و هزینه‌ها (55/5درصد). درمیان موارد پرسش‌نامه بیشترین میزان رضایت به‌نحوه ارتباط و شیوه برخورد کارکنان (31/0=SD و 72/4=M) و کمترین میزان رضایت از هزینه‌های پرداختی در مقابل خدمات مرکز (62/0=SD و 27/3=M) مربوط بود. نتایج حاصل از همبستگی اسپیرمن بین ابعاد ویژه و رضایت کلی به‌طور معناداری مثبت بود (P<0/01) که روایی ساختار پرسش‌نامه را تأیید کرد. در تحلیل بین میزان رضایت از خدمات دریافتی سن، میزان تحصیلات، تعداد اعضای معلول در خانواده، مدت‌زمان رسیدن به کلینیک و وضعیت شنوایی والدین ارتباط معناداری دیده نشد.  نتیجه‌گیری نتایج حاصل از این مطالعه حاکی از آن بود که پرسش‌نامه حاضر روایی و پایایی مناسبی داشت. به‌علاوه به‌نظر می‌رسد والدین از بیشتر زمینه‌هایی که در موارد پرسش‌نامه به آن‌ها پرداخته‌شده و درمجموع از کل خدمات بسیارراضی بودند.

Title: مقاله مروری: مسائل بهداشت باروری‌جنسی در زنان مبتلا به مشکلات جسمی‌حرکتی و ارائه راهکارها: مروری بر متون پژوهشی

http://rehabilitationj.uswr.ac.ir/article-1-2083-fa.html

هدف هدف از این مطالعه، شناسایی مسائل بهداشت باروری‌جنسی زنان با مشکلات جسمی‌حرکتی در پنج حیطه سلامت جنسی، غربالگری بیماری‌های شایع زنان، سلامت مادران، پیشگیری از بارداری ناخواسته و پیشگیری از بیماری‌های مقاربتی است.  روش بررسی در این مطالعه مروری که در سال 1395 انجام شده است، از مقالات نمایه‌شده در پایگاه‌های اطلاعاتی پروکوییست، اس.آی.دی، مگیران، آوید، ایران‌داک، ایران‌مدکس، ساینتس‌دایرکت، کورِنس، اشپرینگر، گوگل‌اسکالر و اسکوپوس استفاده شد. مجموعه مقالات بررسی‌شده شامل ۶۱ مقاله (از 18 کشور مختلف) بود که تحقیقات کیفی و کمّی مربوط به ۱۵ سال اخیر (از سال ۲۰۰۰ تاکنون) را به زبان‌های فارسی و انگلیسی دربرداشت. پژوهش‌هایی که حجم نمونه و نحوه اجرای نامشخصی داشتند یا فقط روی معلولان ذهنی یا مردان انجام شده بود، از روند مطالعه حذف شدند. همچنین مقالاتی که متن کامل آن‌ها دردسترس نبود نیز از مطالعه حذف شدند. یافته‌ها شیوع ۱۰درصدی زنان ناتوان در سنین باروری و افزایش سالانه 5/8میلیون نفری افراد ناتوان، بر ضرورت تلاش برای یافتن راه‌حل‌هایی برای ارائه هرچه بهتر خدمات تأکید دارد. بااین‌حال در این زمینه تحقیقات، به‌ویژه پژوهش‌های کیفی، بسیارمحدود بوده است. نتایج بیانگر آن بود که در بیشتر جوامع، دانش لازم درباره ناتوانی و چگونگی ارائه خدمات بهداشتی به این گروه از افراد کم بوده است. مهم‌ترین علل دسترسی‌نداشتن این جوامع به مراقبت‌های بهداشتی عبارت بود از: مشکلات بیمه و مالی، نداشتن دسترسی فیزیکی به مراکز بهداشتی، نگرش منفی ارائه‌دهندگان خدمت، نبود ِارتباط مؤثر، ساختار فیزیکی غیردوستانه، کمبود تجهیزات، نبودِ نیاز ضروری به انجام آزمون، محدودیت‌های جسمی و شناختی و دسترسی‌نداشتن به تسهیلات. در جوامع اشاره‌شده ارائه خدمات غربالگری پاپ‌اسمیر و ماموگرافی بسیارضعیف بود و بیشتر آن‌ها مشاوره لازم را از پزشک مرتبط دریافت نمی‌کردند و آگاهی پایین و مراقبت‌های ضعیف تنظیم خانواده کاملاً مشهود بود. در ایران نیز افرادی که دچار معلولیت هستند، به‌طور‌چشمگیری مراقبت‌های تنظیم خانواده کمتری دریافت کرده بودند. اغلب این زنان بارداری و زایمان را امری طبیعی می‌دانستند و به بچه‌دارشدن تمایل داشتند. این افراد با توجه به متفاوت‌بودن ارائه خدمات در بارداری و زایمان، تجربه مثبتی از این دوران را بیان می‌کردند؛ اما با افزایش عوارض بارداری و زایمان مواجه می‌شدند. آنان در معرض ابتلا به بیماری‌های مقاربتی بودند و در آن‌ها ارائه مشاوره جنسی ضعیف بود؛ زیرا بیشتر پزشکان تصور می‌کردند که آن‌ها رابطه جنسی ندارند. نتیجه‌گیری با توجه به پایین‌بودن آگاهی و دریافت مراقبت‌های بهداشت باروری‌جنسی در این گروه، آگاه‌سازی جامعه به‌ویژه افرادی که به‌نحوی با معلول ارتباط دارند، از مهم‌ترین برنامه‌هایی است که می‌تواند در درجه اول به شناخت و دستیابی مناسب‌تر به شیوه‌های انطباق خانواده و جامعه با معلول و برعکس منتهی شود و درآینده خدمات توان‌بخشی را نیز بهبود بخشد. همچنین به‌نظر می‌رسد باید به‌دنبال ارائه راهکاری برای افزایش توجه مسئولان به این گروه از افراد جامعه بود. برای رسیدن به این مهم باید نیازسنجی مرتبط صورت گیرد تا براساس آن سیاست‌های بهداشتی لازم برای آن‌ها درنظرگرفته شود و از این طریق بتوان به ارتقای سلامت افراد ناتوان جامعه کمک کرد
